# Streamlining Genetic Diagnosis With Long-Range Polymerase Chain Reaction (PCR)-Based Next-Generation Sequencing for Type I and Type II Collagenopathies

**DOI:** 10.7759/cureus.50482

**Published:** 2023-12-13

**Authors:** Yo Niida, Sumihito Togi, Hiroki Ura

**Affiliations:** 1 Center for Clinical Genomics, Kanazawa Medical University Hospital, Uchinada, JPN; 2 Division of Genomic Medicine, Department of Advanced Medicine, Medical Research Institute, Kanazawa Medical University, Uchinada, JPN

**Keywords:** genetic test, inherited disease, collagen genes, next generation sequencing (ngs), molecular genetic testing

## Abstract

In the practice of clinical genetics, gene testing is usually guided by clinical diagnosis. When dealing with rare diseases, it is often necessary to create new test systems. The handling of a gene with a substantial number of exons poses a challenge both in sequential Sanger sequencing for each exon, and in the setup of capture probes to each exon for next-generation sequencing (NGS). We present very long amplicon sequencing (vLAS), an optimized long-range polymerase chain reaction (PCR)-based NGS method that overcomes this challenge. By utilizing approximately 20 Kb long PCR products and short-read NGS, vLAS is emerging as a highly adaptable and effective solution, especially for genes with numerous exons concentrated in a limited genomic region. Here, we demonstrate vLAS in the analysis of five patients with type I and two with type II collagenopathies. The integration of user-friendly NGS methods into genetic diagnosis enhances the practicality of clinical genetics.

## Introduction

Advances in genetic technologies have made genetic testing more accessible than ever before, with whole exome sequencing (WES) gaining popularity. However, the actual need for daily genetic testing more often revolves around targeted gene analysis rather than comprehensive examination. In the case of rare genetic diseases, it is often necessary to launch new testing systems. When the gene of interest has an extensive number of exons, performing Sanger sequencing on each exon or setting up capture probes for each exon in next-generation sequencing (NGS) becomes challenging. Previously, we used CEL nuclease-mediated heteroduplex incision with polyacrylamide gel electrophoresis and silver staining (CHIPS) technology for mutation screening to streamline the Sanger sequencing process [1,2]. However, the current landscape has shifted towards targeted NGS sequencing [3,4]. There is a growing need for an NGS method that allows easy setup and on-demand analysis of individual genes. Long-range polymerase chain reaction (PCR)-based NGS provides a solution to this challenge. With improved DNA polymerase and high molecular weight DNA extraction methods, we can now reliably amplify approximately 20 kb of specific gene regions from genomic DNA, and library preparation from long PCR products is straightforward. Given that the median size of a human gene on the genome is around 26 Kb [5], in many genes, one or two primer sets can cover the entire gene including the promoter region. We call this method very long amplicon sequencing (vLAS). This approach has particular advantages for genes characterized by numerous exons within a limited region. To illustrate its effectiveness, we present here an analysis of common inherited bone diseases.

Osteogenesis imperfecta (OI) is a genetic connective tissue disorder characterized by bone fragility, with its primary feature being bone fragility. The majority of OI cases result from pathogenic variants in either the *COL1A1* or *COL1A2* gene, which encode for collagen type I alpha chains [6,7]. OI is classified into four types based on clinical severity and radiographic findings: classic non-deforming OI with blue sclerae (Type I), perinatally lethal OI (Type II), progressively deforming OI (Type III), and common variable OI with normal sclerae (Type IV) [8]. As genetic diagnosis advances, it plays a crucial role in predicting the severity of the phenotype based on the genotype [6].

Mutations in the *COL2A1* gene cause a variety of autosomal dominant skeletal dysplasias. The most severe phenotypes, such as achondrogenesis type II, hypochondrogenesis, and Torrance type platyspondylic dysplasia, are associated with neonatal death. Moderate severity phenotypes include spondyloepiphyseal dysplasia congenita (SEDC), Strudwick type spondyloepimetaphyseal dysplasia (SEMD), Kniest dysplasia, spondyloperipheral dysplasia, and Czech dysplasia. Milder forms include early-onset osteoarthritis and Stickler syndrome type I (SSI), the most common type II collagenopathy (1/10,000). Early diagnosis is crucial for appropriate patient care, follow-up, and genetic counseling for affected families [9].

*COL1A1* spans a genomic region of approximately 17.5 kb and contains 51 exons. Similarly, *COL1A2* and *COL2A1 *are approximately 36.7 kb and 31.5 kb and contain 52 and 54 exons, respectively. This paper presents vLAS-analyzed data for patients with OI and SSI.

## Materials and methods

This study was performed at the Center for Clinical Genomics, Kanazawa Medical University Hospital, Uchinada, Japan. Since 2013, we have been contracting out genetic testing for various genetic diseases from in-hospital and out-of-hospital facilities in Japan. A total of seven patients were enrolled, comprising five with OI and two with SSI. Among them, three cases were previously diagnosed using CHIPS screening and Sanger sequencing, while four were newly identified cases (Table 1). Patients six and seven had previously reported CHIPS results [1,2]. All patients were clinically diagnosed by an experts' panel including clinical geneticists, radiologists, orthopedic surgeons, and pediatricians, based on clinical symptoms and bone radiographs. For all participants, genetic testing was conducted following genetic counseling, and written informed consent was obtained from the participating patients or their parents in the study after explanation by the primary physician. This study was conducted in accordance with the Declaration of Helsinki and approved by the Institutional Review Board of Kanazawa Medical University (G161 approved on August 29, 2022).

vLAS was performed as previously reported [10-12]. Briefly, 20 ng of peripheral blood DNA was amplified to approximately 20 kb using the 0.15 micromolar PCR primers listed in Table 1 and KOD Multi&Epi (TOYOBO, Osaka, Japan) to cover the target genes. PCR products were purified using an AMPure XP (Beckman Coulter Life Sciences, San Jose, CA). An NGS library was prepared using an Illumina DNA Prep with Enrichment Kit (Illumina, San Diego, CA), and a 12.5 pM library was loaded on an Illumina MiSeq (Illumina, Inc., San Diego, California, United States) system using a Reagent Nano Kit v2 (500 cycles) (Illumina, Inc., San Diego, California, United States) according to the manufacturer's recommended protocol. Variant calling was performed using GATK's HaplotypeCaller (Version 4.0.6.0) (Broad Institute, Cambridge, Massachusetts, United States) [13], and functional classification of variants was performed using SnpEff (version 4.3t) [14]. The Database of Short Genetic Variations dbSNP (version 151) and ClinVar were used for variant annotation [15,16]. The Integrative Genomic Viewer (IGV, version 2.4.13) (Broad Institute, Cambridge, Massachusetts, United States) was used for visualization [17]. Classification of the pathogenicity of variants was followed by the American College of Medical Genetics and Genomics/American Association of Molecular Pathology (ACMG/AMP) standard guidelines [18]. In patients two and three, novel missense variants were detected in COL1A1. These variants are absent in the general Japanese population according to the Japanese Multi Omics Reference Panel, jMorp (https://jmorp.megabank.tohoku.ac.jp/ last accessed November 20, 2023), and the Genome Aggregation Database, gnomAD (https://gnomad.broadinstitute.org/ last accessed November 20, 2023). Also, this variant has not been registered in ClinVar (https://www.ncbi.nlm.nih.gov/clinvar/ last accessed November 20, 2023). Different missense changes, p.Gly1001Cys, p.Gly731Ala, and p.Gly731Val, of the same amino acid residue, have been reported as pathogenic on ClinVar. Multiple lines of computational evidence support a deleterious effect, and the patient’s phenotypes are highly specific for a disease. These variants are judged to be likely pathogenic according to ACMG/AMP Guidelines. All detected pathogenic variants in this study were registered in ClinVar, with the submission ID SCV004098662-004098668.

**Table 1:** Long-rang PCR primers for COL1A1, COL1A2, and COL2A1. PCR, polymerase chain reaction

Primer set	Primer name	Primer sequence	Primer position (GRCh38.p14)	Product size (bp)
COL1A1 Long	COL1A1_L_F	5'-CCGGGATTTTCAAGAAAGAGATGAGG-3'	50202898_50202873	19061
	COL1A1_L_R	5'-CTGTATGGTATTGCAGGGGAGAAGAGG-3'	50183838_50183864	
COL1A2-A Long	COL1A2_A_F	5'-AGATGGGACAGAGAGTAAGCAGCAAGG-3'	94393807_94393833	20227
	COL1A2_A_R	5'-GTGGTGGAGAAGAGAGGTACGGTATGG-3'	94414033_94414007	
COL1A2-B Long	COL1A2_B_F	5'-CTCAGGGAGTTTCCTTTCAACACAGG-3'	94412774_94412799	19030
	COL1A2_B_R	5'-AACTTGATTTGGCAGGTAGGTGTTCG-3'	94431803_94431778	
COL2A1-A Long	COL2A1_A_F	5'-ATGCTCCTTAAAGGCAGAAAGCTACCC-3'	48009490_48009464	20756
	COL2A1_A_R	5'-ATCCTCCAGAGAACTCCGTTAACATGG-3'	47988735_47988761	
COL2A1-B Long	COL2A1_B_F	5'-GCTAAAGACAAGCTCCATCTCCTGTCC-3'	47991736_47991710	19542
	COL2A1_B_R	5'-ATGCTACTGCCCTCTGATTGATTTGG-3'	47972195_47972220	

## Results

**Figure 1:**
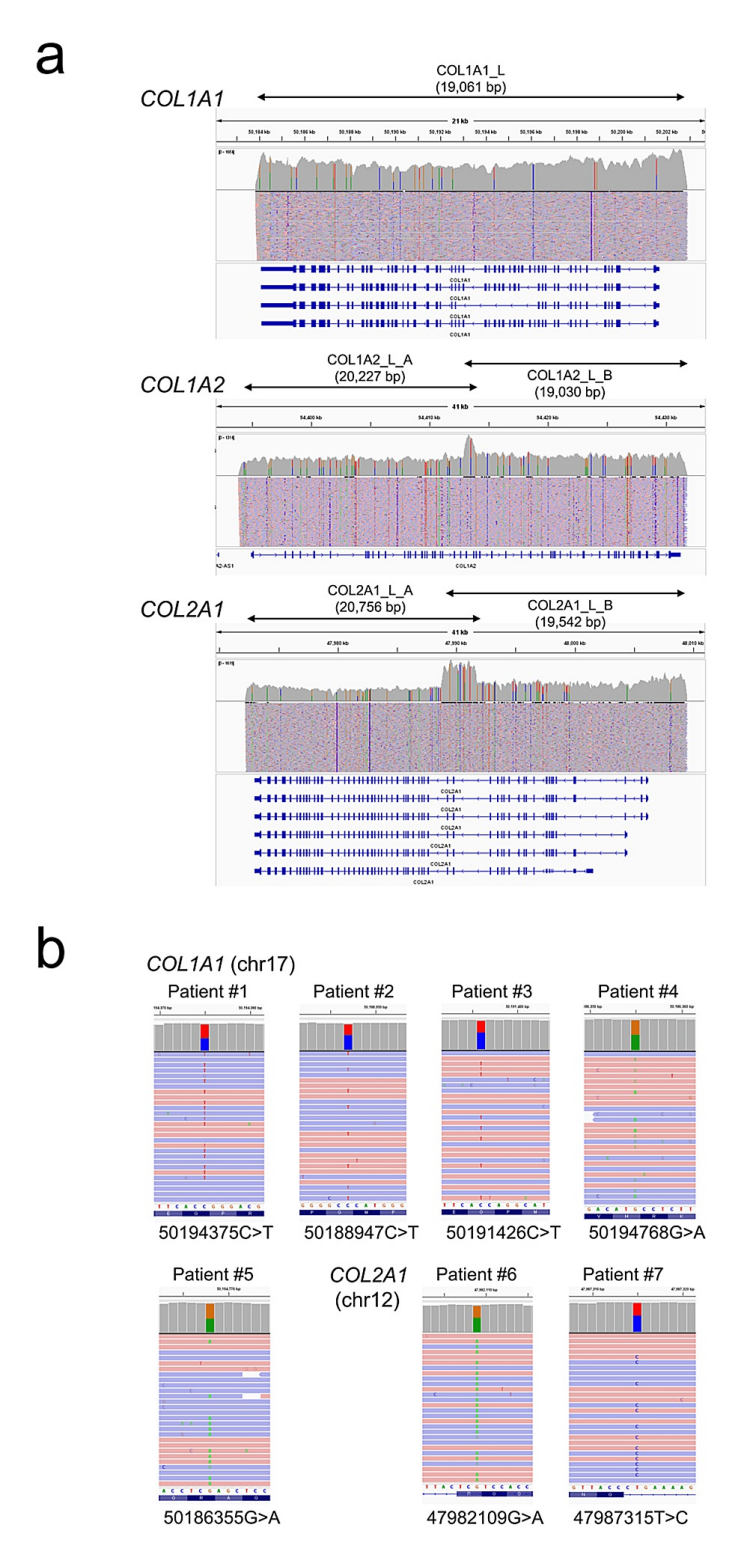
Results of vLAS. (a) Examples of IGV* images of sequencing results for each gene. Whole gene view. (b) IGV image of pathogenic variants of each patient. vLAS, very long amplicon sequencing *Integrative Genomic Viewer (Broad Institute, Cambridge, Massachusetts, United States)

As a result, vLAS was implemented extremely efficiently. Long-range PCR amplification products of COL1A1, COL1A2, and COL2A1 were able to be stably obtained from all patient samples. The IGV image of sequencing results showed that the genomic region of each gene was covered extremely uniformly including all over 50 exons (Figure 1a). Pathogenic variants were detected in all samples, two of which were novel variants (Figure 1b, Table 2).

**Table 2:** Enrolled patients and detected variants. LP, likely pathogenic; NA, not applicable; OI, osteogenesis imperfecta; P, pathogenic; SSI, Stickler syndrome type I * previously analyzed sample, $ reference sequences are NM_000088.4 for COL1A1, and NM_001844.5 for COL2A1, # c.3001G>T p.(Gly1001Cys), % Novel mutations reported by this study first.

#Pt	Age	Phenotype	Gene	#Chr	Pos (hg38)	REF	ALT	HGVS_format^$^	dbSNP_ID	ClinVar
1*	0y2m	OI Type III	COL1A1	Chr17	50194375	C	T	c.1588G>A p.(Gly530Ser)	rs67682641	P
2	0y1m	OI Type IV			50188947	C	T	c.3001G>A p.(Gly1001Ser)	rs72653167^#^	LP^%^
3	0y0m	OI Type II			50191426	C	T	c.2192G>A p.(Gly731Asp)	NA	LP^%^
4	31y	OI Type I			50194768	G	A	c.1414C>T p.(Arg472Ter)	rs72648343	P
5	23y	OI Type I			50186355	G	A	c.3967C>T p.(His1323Tyr)	rs72656344	LP
6*	1y	SSI	COL2A1	Chr12	47982109	G	A	c.2353C>T p.(Arg785Ter)	rs886043410	P
7*	28y	SSI			47987315	T	C	c.1222-2A>G	rs2136577259	P

## Discussion

With advances in DNA sequencing technology, WES and whole genome sequencing (WGS) have become increasingly available. However, in clinical genetic practice, there is often a need for genetic testing focused on individual diseases rather than a comprehensive analysis. Nevertheless, there are limited applications that address this issue, leading to a preference for WES in certain scenarios, including when the target gene has a large number of exons, there are multiple target genes, or the target disease is rare and no specific test is available. The declining cost of WES has further fueled this trend. While capture sequencing methods targeting coding exons are widely used to test individual genes by NGS, the process of setting up new probes is expensive and requires some effort. In addition, the construction of diagnostic panels for specific disease categories makes it daunting to make changes or add new genes as needed. vLAS efficiently addresses these difficulties especially when the number of genes to be tested is limited, and also provides several advantages. First, when analyzing a new gene, genetic testing can be freely performed by simply designing a long PCR primer set for any gene. Second, unlike the capture probe method, there is no hybridization process, so library preparation is easy, there is no off-target generation, and comprehensive analysis including intron regions can be performed [12]. Additionally, vLAS enables the detection of the breakpoint sequences caused by large intragenic deletion [10]. In cases where the gene exhibits mRNA expression in blood, splicing abnormalities due to deep intron variants can also be detected using long-range reverse transcribed PCR (RT-PCR) libraries [10,11].

Nano Kit v2 (500 cycles) reagent is sufficient to run 25 samples with different genes at 100 kb per sample. The average sequence depth is more than 200 and the total cost per sample is less than US$25 including DNA extraction and library preparation [12]. Although vLAS is particularly useful for genes that have a large number of exons in a small region, it can be applied to any gene. In actual clinical practice, testing for specific genes is often required based on clinical diagnosis rather than comprehensive genetic analysis. In such cases, vLAS is a promising option if no test system has been established.

## Conclusions

Streamlining the genetic diagnosis of type I and type II collagenopathies has been achieved with vLAS. vLAS is extremely useful for genetic diagnosis when the target genes can be narrowed down from the clinical diagnosis. It is easy to set up a new test system, and it is possible to detect a wider range of mutations at a low cost. Because of its versatility, vLAS can also be used to diagnose other genetic diseases.
